# Functional Hydrogels for Delivery of the Proteolytic Enzyme Serratiopeptidase

**DOI:** 10.3390/gels10030156

**Published:** 2024-02-20

**Authors:** Katya Kamenova, Anna Prancheva, Stiliyana Stoyanova, Lyubomira Radeva, Ivanka Pencheva-El Tibi, Krassimira Yoncheva, Martin A. Ravutsov, Maya K. Marinova, Svilen P. Simeonov, Simona Mitova, Rumyana Eneva, Maya M. Zaharieva, Hristo Najdenski, Petar D. Petrov

**Affiliations:** 1 Institute of Polymers, Bulgarian Academy of Sciences, 1113 Sofia, Bulgaria; kkamenova@polymer.bas.bg (K.K.); a_prancheva@polymer.bas.bg (A.P.); 2 Open Laboratory on Experimental Micro and Nano Mechanics (OLEM), Institute of Mechanics, Bulgarian Academy of Sciences, Acad. G. Bonchev Str. Block 4, 1113 Sofia, Bulgaria; s_stoyanova@imbm.bas.bg; 3 Roberval Laboratory for Mechanics, Centre de Recherche de Royallieu, Université de Technologie de Compiègne, 60203 Compiegne, France; 4 Department of Pharmaceutical Technology and Biopharmaceutics, Faculty of Pharmacy, Medical University of Sofia, 1000 Sofia, Bulgaria; l.radeva@pharmfac.mu-sofia.bg (L.R.); itibi@pharmfac.mu-sofia.bg (I.P.-E.T.); kyoncheva@pharmfac.mu-sofia.bg (K.Y.); 5 Institute of Organic Chemistry with Centre of Phytochemistry, Bulgarian Academy of Sciences, 1113 Sofia, Bulgaria; martin.ravutsov@orgchm.bas.bg (M.A.R.); maya.marinova@orgchm.bas.bg (M.K.M.); svilen.simeonov@orgchm.bas.bg (S.P.S.); 6 Research Institute for Medicines (iMed.ULisboa), Faculty of Pharmacy, Universidade de Lisboa, Av. Prof. Ga-ma Pinto, 1649-003 Lisbon, Portugal; 7 The Stephan Angeloff Institute of Microbiology, 1113 Sofia, Bulgariarum_eneva@abv.bg (R.E.); zaharieva26@yahoo.com (M.M.Z.); hnajdenski@gmail.com (H.N.)

**Keywords:** serratiopeptidase, citric acid, natural hydrogels, pH sensitive materials, drug delivery, wound healing

## Abstract

Hydrogels are superior wound dressings because they can provide protection and hydration of the wound, as well as the controlled release of therapeutic substances to aid tissue regeneration and the healing process. Hydrogels obtained from natural precursors are preferred because of their low cost, biocompatibility, and biodegradability. We describe the synthesis of novel functional hydrogels based on two natural products—citric acid (CA) and pentane-1,2,5-triol (PT, a product from lignocellulose processing) and poly(ethylene glycol) (PEG-600)—via an environment friendly approach. The hydrogels were prepared via monomer crosslinking through a polycondensation reaction at an elevated temperature in the absence of any solvent. The reagents were blended at three different compositions with molar ratios of hydroxyl (from PT and PEG) to carboxyl (from CA) groups of 1:1, 1:1.4, and 1.4:1, respectively. The effect of the composition on the physicomechanical properties of materials was investigated. All hydrogels exhibited pH-sensitive behavior, while the swelling degree and elastic modulus were dependent on the composition of the polymer network. The proteolytic enzyme serratiopeptidase (SER) was loaded into a hydrogel via physical absorption as a model drug. The release profile of SER and the effects of the enzyme on healthy skin cells were assessed. The results showed that the hydrogel carrier could provide the complete release of the loaded enzyme.

## 1. Introduction

Inflammatory, adhesion, edema, and pain are among the common accompanying post-surgery complications for patients. The effective control and treatment of these post-operative problems are very important for reducing discomfort and achieving faster recovery [1]. Hydrogels are a promising platform for post-operative local treatment due to their hydrophilic nature and biocompatibility. Hydrogels are hydrophilic soft polymeric materials composed of a three-dimensional polymer network which absorbs and retains a large volume of water [2]. Such materials resemble the extracellular matrix (ECM) and are therefore widely used as carriers of drugs, peptides, and proteins or as components for the preparation of protein or enzyme conjugates. Hydrogels are superior wound dressings because they can provide protection and hydration of the wound, as well as the controlled release of therapeutic substances to aid tissue regeneration and the healing process [3,4,5]. Hydrogels can be fabricated from natural and/or synthetic polymers, monomers, and combinations of them. The hydrogel formation involves a process of physical or chemical cross-linking via different routes of bonding/interaction. Indeed, the bonds between the individual chains (junction points) make hydrogels insoluble, while the presence of hydrophilic functional groups such as hydroxyl groups (–OH), carboxyl groups (–COOH), amino groups (–NH_2_), sulphate groups (–SO_3_H), etc., within the network makes the absorption and accumulation of water possible [6,7,8]. The type of hydrogel precursor (monomers, polymers, crosslinking agents) and, specifically, the kind of functional group both have a notable effect on the material properties. Usually, hydrogels obtained from natural precursors are preferred because of their low cost, biocompatibility, and biodegradability [9,10,11]. Particularly, biodegradable hydrogels of high viscoelasticity, softness, and drug loading capacity are widely used in the pharmaceutical and medical fields. Recently, hydrogels for biomedical purposes have been designed to respond to various stimuli, such as pH, temperature, electric field, biological molecules, and ionic strength [7,12,13,14]. pH-sensitive hydrogels have acquired great interest in the controlled delivery of drugs at specific conditions, including pH changes [15,16,17]. The pH of healthy skin is slightly acidic (4.8 to 5.7), but when the skin is damaged and infected, the pH of the wound microenvironment is higher [4].

Citric acid is a multifunctional, water-soluble, non-toxic, natural monomer used to produce pH-sensitive gels. CA comprises one –OH and three –COOH groups, which can be exploited for crosslinking and/or conjugation reactions with other (macro)molecules. An advantage of using CA as a crosslinker is that it has a positive effect on the mechanical properties and stability of the material. CA-based hydrogels can be obtained through a catalyst-free polycondensation reaction including anhydride intermediate formation and the release of water as a by-product at an elevated temperature [10,11,18,19,20]. We recently reported on the preparation of highly elastic super-macroporous cryogels of biodegradable 2-hydroxyethylcellulose, crosslinked with citric acid. The polymer network was formed in bulk at temperatures above 100 °C [21]. Sabzi et al. have synthesized a pH-sensitive antibacterial drug delivery system from poly(vinyl alcohol) (PVA), CA, and Ag nanoparticles [22]. The free carboxylic acid groups of CA, which did not participate in the crosslinking reaction, imparted pH-responsive properties of hydrogel obtained.

Serratiopeptidase (EC number 3.4.24.40) is a proteolytic enzyme, defined as a “super enzyme”, with proven potent anti-inflammatory activity. This enzyme was first isolated from enterobacteria *Serratia* sp., which exist in the gut of the silkworm [23,24]. Serratiopeptidase (SER), also known as serralysin, serratiaprotease, and serrapeptase, is used in various fields of medicine such as surgery, orthopaedics, otorhinolaryngology, gynaecology, and dentistry as an anti-inflammatory, anti-endemic, and analgesic agent. Serratiopeptidase is an alternative to traditional non-steroidal anti-inflammatory drugs to treat inflammation. It is effective in reducing swelling and edema and metabolizing scar tissues in the body and helps in wound healing. SER acts as an anti-inflammatory agent by regulating inflammatory cytokines and also accelerates the healing process due to its unique property of breaking down fibrous tissue around the injured site without damaging living tissue [25,26,27,28]. Singh and Singh developed effective biocompatible PVA hydrogel loaded with SER and gentamicin for effective and complete wound healing [29]. The prepared hydrogel provides sustained release of the two loaded substances at the wound surface. A local gel formulation of SER with anti-inflammatory effects was studied from Nirale and Menon [30]. In vitro and in vivo tests showed satisfactory anti-inflammatory activity and inhibition of ear edema in rats.

In this study, we report the synthesis of novel pH-sensitive hydrogels from two natural products (CA and PT) and PEG-600 as carriers of SER. PT is a renewable polyfunctional compound obtained from the furfural processing [31]. Recently, our research group has fabricated biocompatible and biodegradable nanogels for drug delivery, based on PT and CA, via precipitation polymerization through a Steglich esterification reaction with N-ethyl-N′-(3-dimethylaminopropyl) carbodiimide (EDC) and a 4-dimethyl aminopyridine (DMAP) catalyst system in an organic solvent [32]. In the present work, the crosslinking of PT and PEG with CA was achieved by a facile, environment friendly process at an elevated temperature in the absence of any solvent. To the best of our knowledge, hydrogels from PT (PEG) and CA have not been reported as carriers for enzyme delivery. Thus, in the present study, SER was loaded as a model enzyme with the aim of studying such a possibility. Hydrogels were obtained from PT, PEG, and CA blends of three different compositions, with molar ratios of hydroxyl to carboxyl groups of 1:1, 1:1.4, and 1.4:1, respectively. The influence of the composition on the gel fraction (GF) yield, swelling degree (SD), and elastic properties were assessed. Next, SER was loaded in the hydrogel and the release profile and effect of SER on healthy cells were evaluated.

## 2. Results and Discussion

### 2.1. Synthesis of Hydrogels

**Figure 1:**
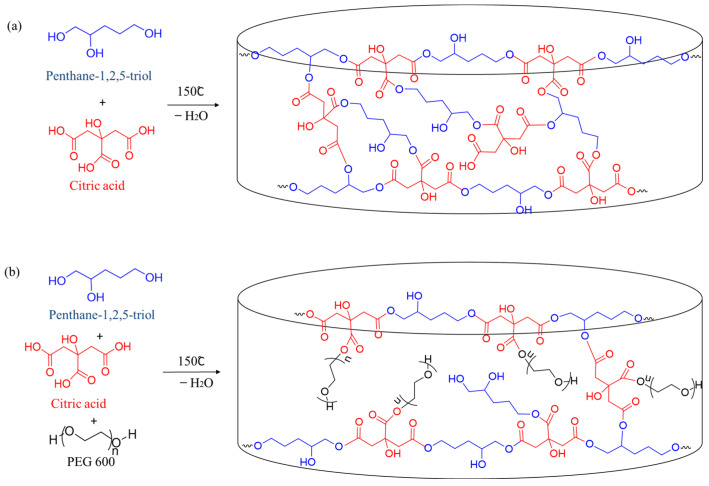
Schematic illustration of the synthesis of hydrogels based on (**a**) pentane-1,2,5-triol and citric acid and (**b**) pentane-1,2,5-triol, citric acid, and PEG-600.

Two types of pH-sensitive hydrogels were obtained through a polycondensation reaction of CA and PT or CA, PT, and PEG-600 at 150 °C under vacuum in the absence of solvent. The polymer network was formed via reactions of CA-based anhydride intermediates with hydroxyl groups from PT and PEG, which lead to the formation of ester bonds (Figure 1).

First, CA and PT were mixed and homogenized with stirring at 80 °C at a molar ratio corresponding to an equal number of –OH and –COOH groups (–OH/–COOH molar ratio 1:1). Then, the reaction mixture was placed in Teflon dishes and the polycondensation crosslinking reaction was carried out in a vacuum oven at 150 °C and 0.04 MPa for 18 h, with continuous removal of water from the reaction mixture. Finally, the resulting gels were purified from unreacted substances via extraction with distilled water. In the next stage, PEG-600 was incorporated into the polymer network to modify the properties of the hydrogel. PEG is a non-immunogenic, non-toxic, biocompatible polymer which has two terminal hydroxyl groups [33]. Hydrogels were obtained from three different compositions of the reaction blend with molar ratios of –OH (PT + PEG) to –COOH (CA) groups of 1:1, 1:1.4, and 1.4:1. The amount of PT was kept constant in all mixtures, while the amount of PEG and CA was varied.

**Figure 2:**
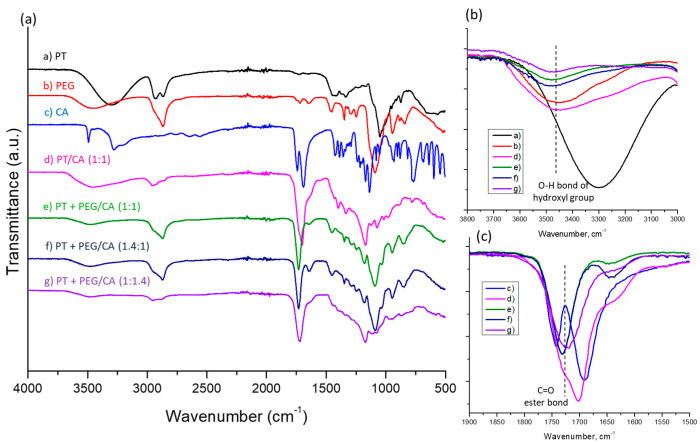
FTIR spectra of the precursors and corresponding hydrogels of different compositions (**a**). FTIR spectra in the 3800–3000 cm^−1^ range associated with the hydroxyl group (**b**). FTIR spectra in the 1900–1500 cm^−1^ range associated with the carboxylic group and ester bond formation (**c**).

The formation of ester bonds within the polymer hydrogels was confirmed via Fourier transform infrared spectroscopy. Figure 2 shows the FTIR spectra of PA, CA, PEG, and the formed polymer networks at different molar ratio of reagents. The FTIR spectra of PT and PEG-600 show identical characteristic peaks at 3500–3200 cm^−1^, assigned to the stretching vibration of O–H bonds; 2970 and 2820 cm^−1^, corresponding to the stretching of C-H bonds; and 1050–1000 cm^−1^, attributed to the C–O stretching of the alcohol [32,34]. CA exhibited characteristic signals at 3500–3000 cm^−1^, from stretching vibrations of O-H bonds and C–H bonds; 1750 and 1690 cm^−1^, corresponding to C=O stretching of the carboxylic groups of aliphatic carboxylic acid; 1180–1100 cm^−1^, correlated with the C–O bonds; and 780 cm^−1^, ascribed to CH_2_ rocking [35]. The FTIR spectra of hydrogels have two new intensive bands which are typical for polyesters [32,36]. The appearance of stretching vibrations of C=O groups at 1726 cm^−1^ (Figure 2c) and twisting vibrations of CH_2_ groups at 1192 cm^−1^ indicates the successful formation of ester bonds between –OH groups of PT and PEG and –COOH groups of CA [22,32]. Furthermore, the intensity of the peaks associated with –OH and –COOH groups also decreased, which means that a part of the functional groups was consumed in hydrogel formation. Increasing the CA fraction decreased the intensity of peaks assigned to –OH groups and the number of free hydroxyl groups in the hydrogels, respectively (Figure 2b).

### 2.2. Effect of Reagents Composition on the Gel Fraction Yield and Swelling Degree

**Figure 3:**
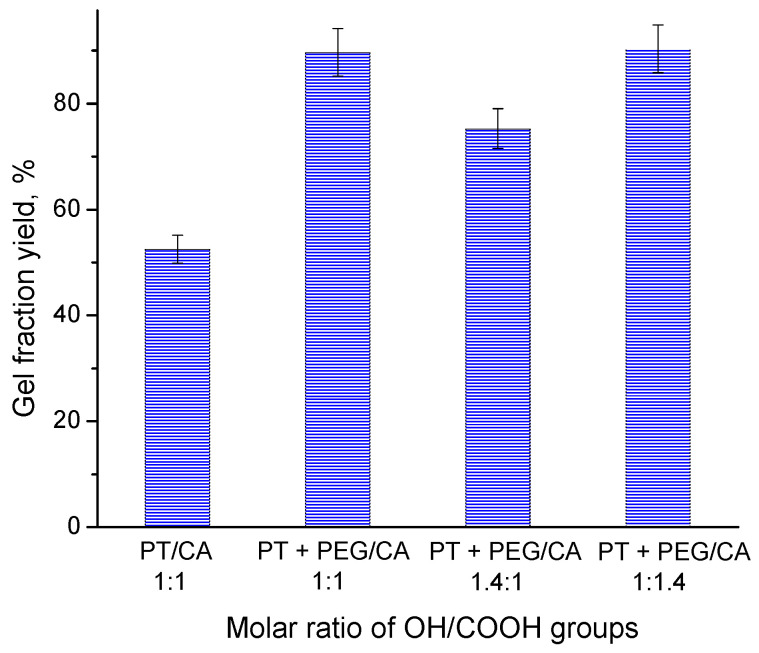
Gel fraction yield of hydrogels obtained from PT, CA, and PEG at different molar ratios of the functional groups.

Gel fraction yield is a measure of the effectiveness of the crosslinking reaction that occurs between the reagents during the formation of the hydrogel network. Generally, the high values of GF indicate a high conversion of monomers/crosslinking reagents, affording a dense polymer network. This, in turn, lowers the swelling capability of the hydrogel due to a reduced storage matrix volume for absorbed water [37]. Figure 3 shows the influence of reagents composition on the GF yield of the hydrogel. The GF yield of hydrogel obtained from pentane-1,2,5-triol and citric acid (molar ratio of functional group 1:1) was relatively low (52%). At first glance, this result is unexpected because each reagent has three reactive groups, which should favor an effective monomer conversion. Bearing in mind that the reaction was carried out in bulk, one possible reason for such low reaction efficiency might be the limited interaction between active species due the steric hindrance of bulky molecules. Adding a relatively longer oligomer comprising two primary –OH end groups (PEG-600) to the initial reagents resulted in a notable increase in GF yield. This means that the flexible PEG molecules were incorporated into the polymer network and facilitated the reaction process. On the other hand, the gels synthesized at an equal molar ratio of the functional groups and an excess of the –COOH groups achieved a GF yield of 90%. Consequently, the higher concentration of CA in the reaction mixtures has a positive effect on the cross-linking process. This influence of the crosslinking agent (citric acid) content on the gel fraction yield is in accordance with a previous study [21].

**Figure 4:**
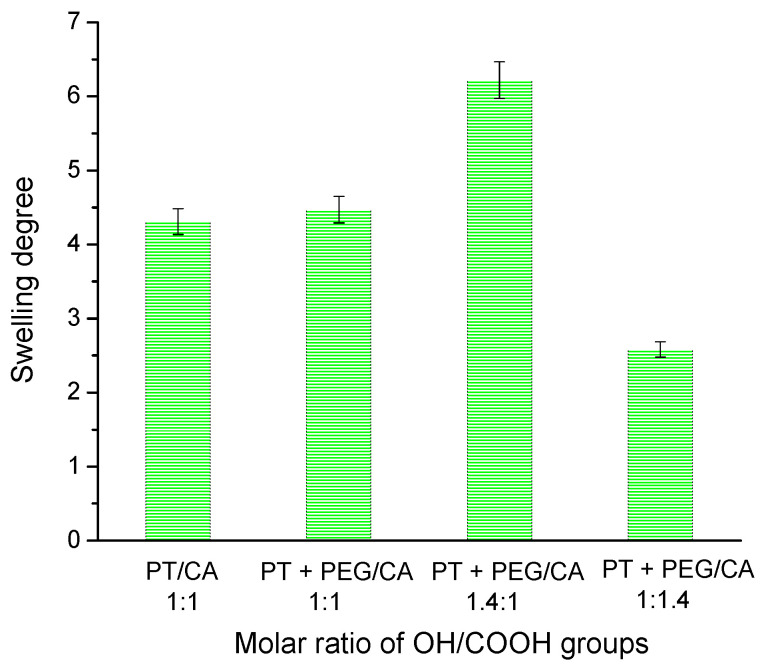
The swelling degree of hydrogels, obtained from PT, CA, and PEG at different molar ratios of the functional groups in distilled water.

First, the SD values of the purified hydrogels were determined after incubating the freeze-dried samples in distilled water until constant mass (24 h). The SD values depended on sample composition and GF yield, respectively (Figure 4).

It is known that the high crosslinking density leads to the low swelling capability of the hydrogel due to the reduced storage matrix volume for absorbed water [35]. In our experiments, the SDs of the hydrogels obtained with the same molar ratio of carboxyl to hydroxyl groups had approximately the same values. The three samples comprising PEG showed a composition-dependent degree of swelling. The higher the molar ratio of OH/COOH groups, the higher the SD. Obviously, the hydrogel with the highest content of CA exhibited a higher degree of crosslinking, which reduced its ability to absorb water.

### 2.3. Dynamic Rheological Studies

**Figure 5:**
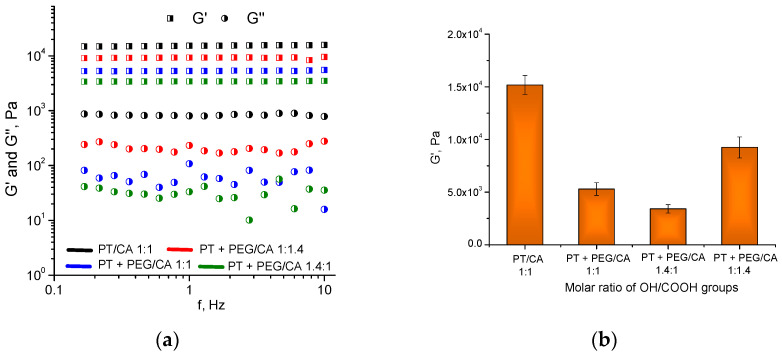
Variation in (**a**) storage and loss moduli as a function of the oscillatory frequency; (**b**) storage modulus as a function of the molar ratio of OH/COOH groups for different hydrogels obtained from PT, CA, and PEG.

The viscoelastic properties of hydrogels were studied via rheological measurements. The measurements of storage (G′) and loss (G″) moduli, which characterize the elastic and viscous components of the hydrogel, respectively, aimed to assess the strength of the materials obtained at different compositions [38]. All hydrogels developed in this study exhibited a storage modulus an order of magnitude higher than the loss modulus (Figure 5a).

These results mean that the elastic response of the gel to the applied shear stress considerably exceeds the viscous one. In addition, G′ and G″ were nearly independent of frequency, which is a typical behavior of highly elastic hydrogel materials. It should be noted that the hydrogel obtained from pentane-1,2,5-triol and citric acid has the highest storage modulus and strength, respectively (Figure 5b). The incorporation of PEG-600 into the polymer network reduced the storage modulus as compared to the PT/CA hydrogel. On the other hand, the three hydrogels containing PEG-600 showed composition-dependent mechanical properties. The material with the lowest fraction of PEG-600 (–OH/–COOH groups 1:1.4) has the highest G′ and vice versa. The amount of absorbed water appears to significantly affect the elastic properties of these hydrogels as materials with a lower degree of swelling possess a larger storage modulus.

### 2.4. pH-Sensitive Properties and Hydrolitic Degradation of Hydrogels

**Figure 6:**
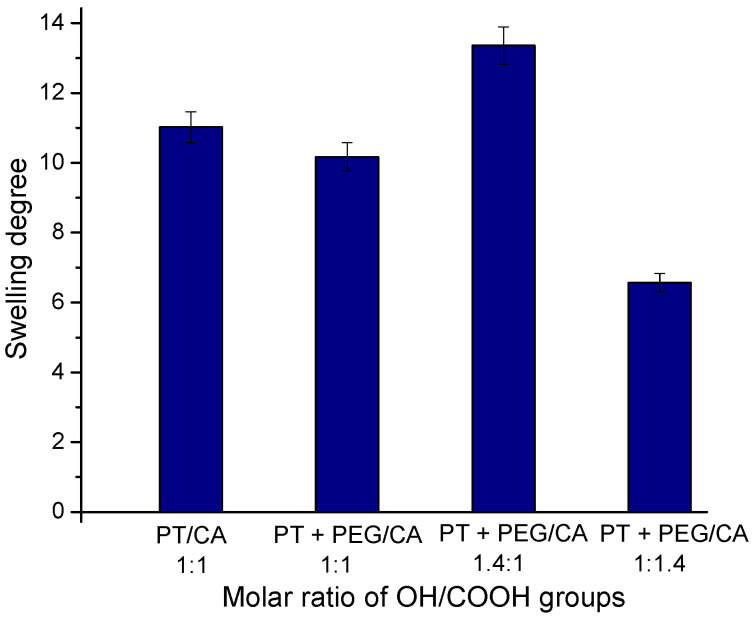
pH-dependent swelling of hydrogels obtained from PT, CA, and PEG at different molar ratios of the functional groups in NaOH solution (pH 12).

The swelling behavior of anionic hydrogels is affected by the dissociation of anionic pendant groups (e.g., –COOH) when the pH of the surrounding aqueous media goes above the acid dissociation constant (pKa). At such conditions, the functional groups tend to lose protons, making the hydrogel negatively charged. The swelling of the charged hydrogel is then triggered by the osmotic pressure induced by the electrostatic repulsion [17]. The pH-sensitivity of the developed hydrogels was demonstrated by a simple experiment involving the exchange of distilled water for sodium hydroxide solution as a medium (pH = 12). The results, given in Figure 6, show that all hydrogels swell by more than two times in an alkaline environment than in pure water. We assume that the dissociation of the free carboxyl groups at pH 12 is complete and that all of them are deprotonated in the basic solution. This causes strong electrostatic repulsion of the polymer segments in the hydrogel the network and increases the swelling ability of material. It should be mentioned that the SDs of the hydrogels in acetate buffer (pH 3.8) were identical with those for distilled water.

Considering the physicomechanical properties of the materials described above, we selected for loading with SER the hydrogel obtained from PT, PEG, and CA at a OH/COOH groups molar ratio of 1:1. This hydrogel combines high GF yield, relatively good elastic properties, and high swelling ability, which favors the entrapment of larger amounts of enzyme.

The long-term stability of the blank hydrogel in distilled water was assessed. The swollen gel was kept in the medium for 30 days at room temperature. The gel did not change its size and shape in the first 5 days. However, in the next 25 days, the material gradually lost its integrity and the mass of the gel (determined in dry state) decreased by 65%. Most probably, the hydrogel decomposition was due to the hydrolysis of ester bonds of the polymer network.

### 2.5. Loading of Hydrogels with Serratiopeptidase, In Vitro Release and Cytotoxicity Studies

Before loading, the proteolytic activity of SER was determined in three different concentrations. The results are shown in Table 1.

**Figure 7:**
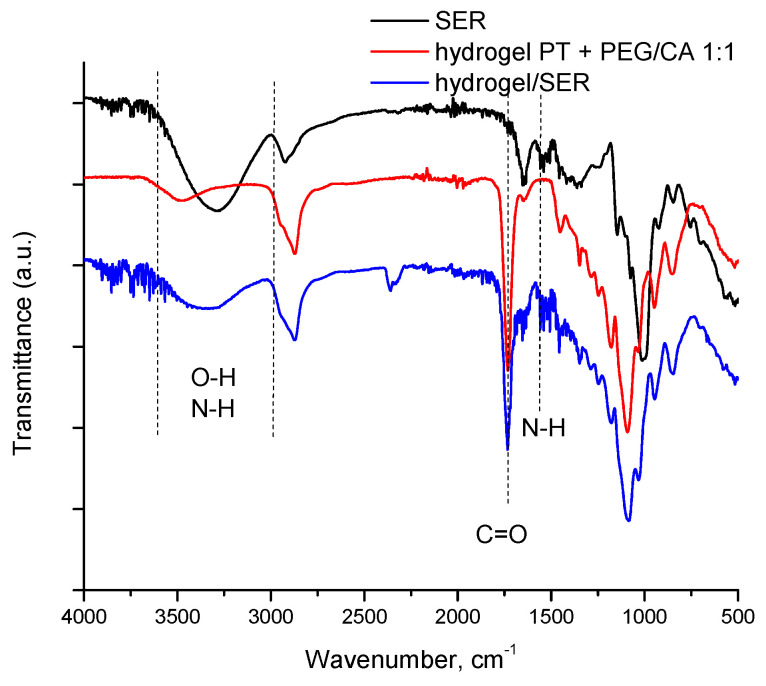
FTIR spectra of serratiopeptidase (black line), blank hydrogel (PT + PEG/CA 1:1) (red line), and hydrogel (PT + PEG/CA 1:1) loaded with serratiopeptidase (blue line).

Next, SER was loaded into the selected hydrogel via physical adsorption. The freeze-dried hydrogel was placed in an aqueous solution of the enzyme (10 mg/mL) until it absorbed the liquid and swelled. The ratio between the enzyme and the gel was 1:25 (*wt*/*wt*). We assume that relatively weak and reversible interactions (i.e., hydrogen bonds, van der Waals forces, electrostatic interaction, etc.) occurred between functional groups of the enzyme and hydrogel material. This is a facile and cheap procedure for the reversible immobilization of enzymes into hydrogels, excluding any covalent immobilization [39,40,41]. The successful loading of SER into the hydrogel was confirmed by FTIR analysis (Figure 7). The main peaks of hydrogel were observed at approximately 3300 cm^−1^ (O–H bond), 2870 cm^−1^ (C–H bond), and 1730 cm^−1^ and 1192 cm^−1^ (from ester), while the vibrations at 3500–3000 cm^−1^ (O–H and N-H), 2900 cm^−1^ (C–H bond of the alkyl chains), and 1655 cm^−1^ and 1545 cm^−1^ (amide I) are assigned to SER. The peak at 1455 cm^−1^ is due to the N-H stretching vibrations present in the amide linkages of the enzyme. 

**Figure 8:**
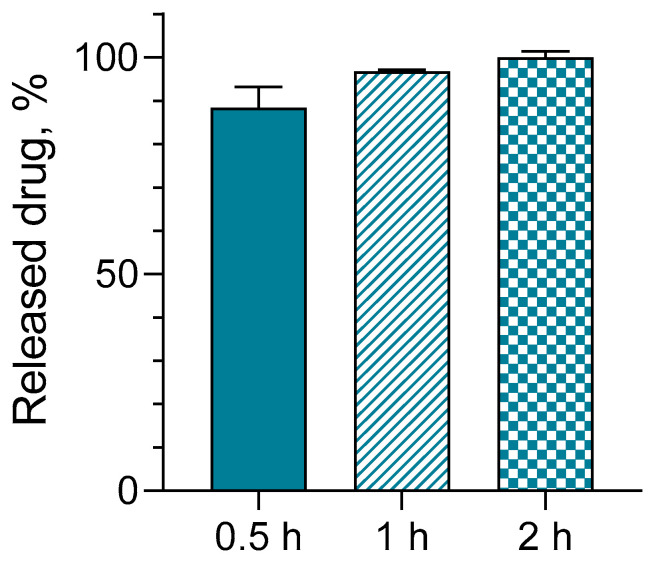
In vitro release test of serratiopeptidase from the gel in medium with pH of 7.4.

Serratiopeptidase is known to possess a wound healing effect due to its potential to degrade abnormal exudates and proteins and to dissolve dead tissue. Moreover, the enzyme could also improve the absorption of fragmented products through the blood and lymph [25,26,28]. Thus, aiming to evaluate the possible skin administration of the developed gel formulation, the in vitro release of the model enzyme was conducted at 32 °C. Considering that the pH of the damaged skin is higher than that of healthy skin (pH ~5), the in vitro release test was performed in a slightly neutral buffer with a pH of 7.4 [42,43]. The results under these conditions showed the fast and complete release of the enzyme for 2 h (Figure 8). As can be seen, approximately 89% of the enzyme was released for the first 30 min, which was probably due to its hydrophilic properties and the very weak interactions between the enzyme molecules and hydrogel carrier. As the isoelectric point of SER is around pH 5.0, we assume that in distilled water (the pH of the dissolved SER in water was ~6.2), most of the amino groups of SER are protonated and some of the carboxyl groups are deprotonated. Therefore, during the loading step, some weak electrostatic interactions are possible between the C=O groups present in the gel and the N–H groups of SER. In the phosphate buffer (pH 7.4), more carboxyl groups become deprotonated, which leads to the generation of carboxylate anions both in SER and in the gel (due to its pH sensitivity). This can lead to electrostatic repulsion and rapid release of the enzyme. 

Considering that polymer carriers containing carboxyl groups can inhibit the activity of enzymes [44], we evaluated the activity of SER after its release from the hydrogel. Unfortunately, proteolytic activity of SER in phosphate buffer (pH 7.4) was found to decrease significantly (~25% of the value before enzyme loading). Since protease enzymes have bivalent cations like zinc and calcium as essential co-factors within their structure, the loss in enzyme activity can be due to some competitive/noncompetitive interactions [30]. It is also known that calcium can protect the enzyme from autolysis, and by introducing additional amounts of Ca^2+^ ions, the activity of SER can be increased [45]. Indeed, our experiments revealed that the enzyme activity can be increased by two times by adding Ca^2+^ to the buffer (SER/CaCl_2_ mass ratio1:1). This is attributed to the fact that calcium ions can keep the enzyme more stable as seven Ca^2+^ ions are required to facilitate the folding of SER. These results suggest that the use of a buffer containing Ca^2+^ ions to dissolve SER before the loading of the enzyme into the hydrogel is more beneficial than pure water.

**Figure 9:**
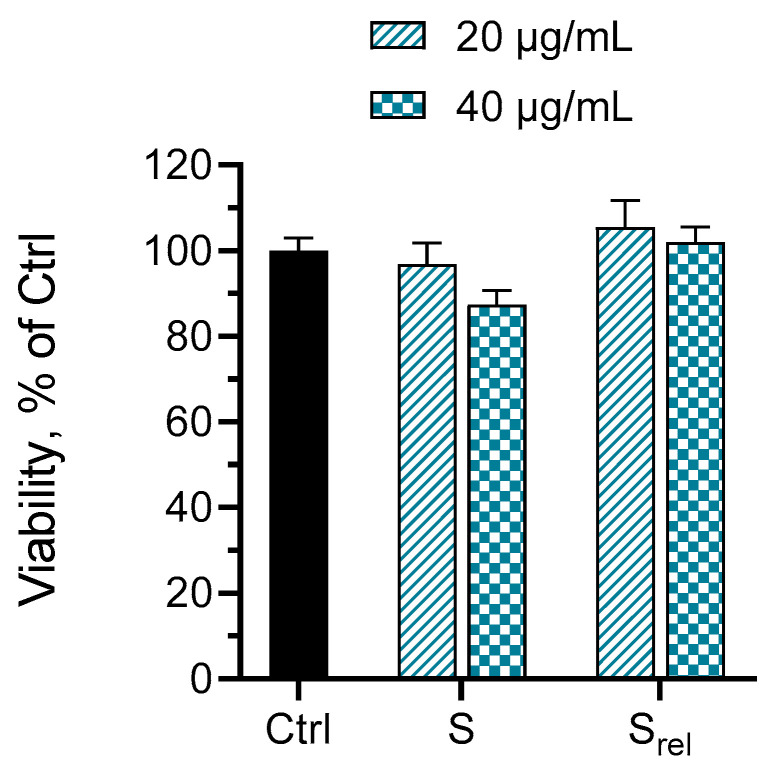
Viability of human keratinocytes (HaCaT) after treatment with a standard solution (S) and a sample of the released (after 1 h of dissolution test, Srel) serratiopeptidase.

Further, a preliminary study aiming to examine the idea for skin administration was performed. Keratinocytes are the most dominant constituents of skin cells and play an important role in the wound healing process. They could activate the immune cells as well as help fight exogenous pathogens. Keratinocytes also stimulate fibroblasts to produce growth factors, which leads to keratinocyte proliferation [46,47,48]. In view of this, we evaluated the cytotoxic effect of the standard solution and the released-from-the-gel-system enzyme on human keratinocytes HaCaT (Figure 9). The results showed that the standard and the released sample of the enzyme did not provoke any cytotoxic effects on HaCaT cells (no statistical difference vs. the control group). This preliminary assessment showed that the incorporated-into-the-gel enzyme has the potential for further evaluation of its pharmacological potential in the wound healing. In addition, the properties of the newly developed functional hydrogel carrier can be modified to change the release rate of the enzyme, including the use of micro- and nanoparticles [29]. Therefore, more experiments focusing on the key factors that may influence SER activity in the wound healing process are needed in order to confirm the benefit of the developed system.

## 3. Conclusions

Novel hydrogels were successfully prepared via an environmentally friendly approach based on the polycondensation reaction of the natural products pentane-1,2,5-triol and citric acid and polyethylene glycol in the absence of a solvent. Adding PEG-600 to the PT/CA blend enhanced the crosslinking efficiency, and the gel fraction yield of the resulting three-component hydrogels was up to 90%, depending on the composition. Generally, the density of the polymer network and the hydrogels’ properties were affected by the molar ratio of hydroxyl (PT + PEG) to carboxyl (CA) groups. The higher the CA fraction, the higher the storage modulus and the lower the swelling degree. All hydrogels exhibited pH-sensitive behavior and swelled more than two times in an alkaline medium than in distilled water and acidic buffer. In addition, the material degraded gradually in water via the hydrolysis of ester groups and lost 65% of its mass within 30 days. It was demonstrated that the model enzyme SER can be loaded in the hydrogel via physical absorption. Preliminary test with healthy skin cells showed that the incorporated-into-the-gel-enzyme has the potential for further evaluation of its pharmacological potential in wound healing.

## 4. Materials and Methods

### 4.1. Materials

Citric acid (99.5%, Sigma-Aldrich, FOT, Sofia, Bulgaria), poly(ethylene glycol) (PW 600 g mol^−1^; Sigma-Aldrich, FOT, Sofia, Bulgaria), and phosphate buffer (Sigma-Aldrich, FOT, Sofia, Bulgaria) were used as received. Pentane-1,2,5-triol was synthesized according to a procedure described elsewhere [49]. Human keratinocytes HaCaT were obtained from CLS Cell Lines Service, 300493 (Eppelheim, Germany). Foetal bovine serum and anhydrous acetic acid were purchased from Sigma Chemical Co. (Darmstadt, Germany).

### 4.2. Extraction, Purification and Proteolytic Activity Assay of Serratiopeptidase Enzyme

Capsules (Doctor’s Best, Tustin, CA, USA) with their compositions indicated on the label—Serrapeptase enzyme and Serrateric™ (maltodextrin, tricalcium phosphate, calcium stearate, microcrystalline cellulose)—were dissolved in distilled water (for maximum purity after recovery by lyophilization) and centrifuged at 9000 rpm for 15 min. The supernatant, containing the dissolved enzyme, was separated. The sediment was washed with distilled water three times and centrifuged to maximize enzyme recovery. Purification and concentration were carried out on an Amicon device with Biomax^®^ 50 kDa ultrafiltration discs (EMD Millipore Corporation, Billerica, MA, USA) and a Diaflo PM 30 membrane (Amicon Corporation, Lexington, MA, USA). The resulting concentrated enzyme was sterile-filtered through a 0.22 µm syringe filter (Millipore) and lyophilized. The enzyme was tested for proteolytic activity by the Sigma’s Non-specific Protease Activity Assay—Casein as a Substrate [50]. The method is based on the enzymatic hydrolysis of casein by SER to tyrosine, whose concentration is determined via UV spectroscopy (Thermo Scientific, Waltham, MA, USA) at 660 nm. One unit of protease activity was defined as micromoles of tyrosine released for 1 min under standard conditions using casein as a substrate. Units per milliliter were defined with the following equation:

(1) $$\frac{\begin{array}{c} \mathrm{Units} \end{array}}{ml}=\frac{µmol\;tyrosine\;equivalents\;released\times total\;volume\;of\;assay\left(ml\right)}{volume\;of\;sample\left(ml\right)\times time\;of\;assay\left(minutes\right)\times volume\;used\;in\;colorimetric\;determination\left(ml\right)}.$$

### 4.3. Synthesis of Hydrogels

Hydrogels based on pentane-1,2,5-triol, citric acid, and poly(ethylene glycol) were prepared by a polycondensation crosslinking reaction in the absence of solvent. The hydrogels were obtained at three different compositions of the reagents, with a molar ratio of hydroxyl from PT and PEG-600 to the carboxyl groups (from CA) of 1:1, 1.4:1, and 1:1.4, respectively. The amount of PT (0.5 g) was kept constant in all mixtures, while the amount of PEG-600 (2.4 g; 1.68 g; and 1.6 g) and CA (1.26 g; 0.76 g; and 1.26 g) varied accordingly. The calculated amounts of reagents were weighed on an analytical balance, blended, and heated with the aid of a magnetic stirrer at 80 °C. Gentle stirring for 3 h was applied to obtain a homogeneous solution. Next, 8 portions of 1 mL from each mixture were quickly placed in Teflon molds, which were put in a vacuum oven at 150 °C and 0.04 MPa for 24 h. One of the hydrogels was synthesized from PT (1 g) and CA (1.59 g) at a molar ratio of functional OH/COOH groups 1:1.

### 4.4. Determination of Gel Fraction Yield and Swelling Degree

All hydrogels were immersed in distilled water (100 mL) and kept for 6 days at 25 °C to remove the unreacted substances. Water was exchanged four times. The mass of swollen gels and dried gels (by lyophilization) were then weighed and the GF yield and SD were determined as follows:

(2) $$GF\left(\%\right)=\frac{mass\;of\;dried\;hydrogel}{initial\;mass\;of\;reagents}\times 100,$$

(3) $$SD=\frac{mass\;of\;swollen\;hydrogel}{mass\;of\;dried\;hydrogel}$$

The experiments were conducted for each hydrogel and the given values are an average of 8 samples.

### 4.5. Hydrogel Swelling at Different pH of Media

The swollen-in-distilled-water hydrogels were transferred in basic (sodium hydroxide solution with pH = 12) and acidic (acetate buffer with pH = 3.8) media and kept for 24 h. The acidic buffer was prepared by mixing sodium acetate (4.08 g) and acetic acid (18.89 mL) with distilled water in a 250 mL volumetric flask. To prepare the sodium hydroxide solution, 0.1 g of sodium hydroxide is added to 250 mL of distilled water. After homogenization of the solution, the pH is measured. The swollen gels were weighed, frozen, and then lyophilized. SD was calculated using the equation given above.

### 4.6. Hydrolytic Degradation of Hydrogel

Pre-weighed freeze-dried hydrogels were immersed in distilled water and kept in the media for 30 days at room temperature. Next, the remaining material was collected, freeze-dried, and weighed. The mass change (MC) was determined gravimetrically as follows:

(4) $$MC\left(\%\right)=\frac{final\;mass\;of\;gel-innitial\;mass\;of\;gel}{initial\;mass\;of\;gel}\times 100.$$

### 4.7. FTIR Spectroscopy

The FTIR spectra of pentane-1,2,5-triol, PEG-600, citric acid, and freeze-dried hydrogels were recorded from 600 to 4000 cm^−1^ hydrogels using an FTIR spectrometer (IRAffinity-1, Shimadzu, Kyoto, Japan) with ATR.

### 4.8. Dynamic Rheological Measurements

Dynamic rheological measurements of hydrogels were performed using a HAAKE RheoStress 600 rheometer (Thermo Fisher Scientific, Waltham, MA, USA) with a parallel plate sensor system (20 mm diameter) and Peltier temperature controller. Storage (G′) and loss (G″) moduli were determined in the 0.03–10 Hz frequency range at a constant shear strain, γ = 0.005, which was inside the linear viscoelastic regime. Three runs of each hydrogel type were performed at 25 °C.

### 4.9. Loading of Hydrogels and In Vitro Release Test

The freeze-dried gel was placed in an aqueous solution of the enzyme (10 mg/mL) until it absorbed the liquid and swelled. The mass ratio between the enzyme and the gel was 1:25.

### 4.10. In Vitro Release Test

An in vitro release study was conducted in a buffer with a pH value of 7.4 at a temperature of 32 °C. The gel loaded with the enzyme was placed in 10 mL of the buffer and gently shaken (IKA Labortechnik HS-B20, Staufen, Germany). At predetermined time intervals (30 min), samples of 1.5 mL were taken from the release medium and the same volume of fresh medium was returned. The concentration of the released enzyme was determined via high-performance liquid chromatography (HPLC). The chromatographic procedure was carried out with an HPLC system (UltiMate Dionex 3000 S Chromeleon 7.2 SR3 Systems, Thermo Fisher Scientific Inc., Waltham, MA, USA). The separation was achieved with Column Luna (Phenomenex) C18, 250 × 4.60 mm, particle size 5 μm, and Diode Array Detector. The isocratic mobile phase was prepared by mixing filtered and degassed acetonitrile and water (70:30 *v*/*v*) and 0.1% anhydrous acetic acid. The detection wavelength was set at 227 nm, the column temperature was 25 °C, and the flow rate about 1.0 mL/min.

### 4.11. In Vitro Cytotoxicity Studies

The cytotoxicity of a standard solution of the enzyme and a sample from the release test at end of the 1st hour was evaluated on human keratinocytes HaCaT cells. The cells were maintained in culture medium DMEM (DMEM-HPA, Capricorn^®^, Ebsdorfergrund, Germany) supplemented with 10% fetal bovine serum and 4 mM L-glutamine and incubated at standard conditions of 5% CO_2_, 37 °C, and maximal humidity. They were seeded in 96-well plates in a starting density of 0.13 × 10^6^ cells/mL under sterile conditions (Laminar Air Flow Telstar Bio II Advance, Spain) and incubated for 24 h until entering the log-phase of the growth curve. Further, the cells were treated for 24 h with the standard solution and the sample from the release test (both in two dilutions: concentrations of 20 and 40 µg/mL). For the control group, only culture medium was used. The cell viability was determined using an MTT assay [51] according to ISO 10993-5-2009 [52]. Then, the cells were incubated with the MTT dye (0.05 mg/mL final concentration) for 2 h at 37 °C. After removing the culture medium, the formazan crystals formed were dissolved in isopropyl alcohol containing 5% HCOOH (Chimspektar OOD, Sofia, Bulgaria). The absorbance was measured at λ = 550 nm (reference filter 690 nm) against a blank solution.

### 4.12. Statistical Analysis

All experiments were performed in triplicate and the results are presented as mean values ± (SD). GraphPad Prism version 8.0.0 for Windows, GraphPad Software (Dotmatics, San Diego, CA, USA) was used for the statistical analysis of the data from the cell viability assay. One-way ANOVA test with Dunnett’s post-test was applied for comparison between the serratiopeptidase-treated groups and the control group.

## Figures and Tables

**Table 1:** Proteolytic activity of SER at three concentrations.

Concentration of SER (mg/mL)	U/mL
0.1	0.36
0.25	0.88
0.5	1.72

## Data Availability

The data presented in this study are openly available in article.
